# Monthly Digest

**Published:** 1889-06

**Authors:** 


### MONTHLY DIGEST.

FOR JUNE.

### The Trend of Dental Thought as Presented by Our Contemporaries.

*April Review, Western Journal, International, Southern and Dental Record; May Archives, Items, Cosmos, Ohio Jaurnal.*

### OPERATIVE DENTISTRY.—FILLING, METHODS, AND MATERIALS.

### ANNEALING GOLD.

In reply to questions from Dr. Harlan, Dr. G. V. Black and Dr. W. C. Barrett furnish the *Review* (Jan. and April issues) two valuable papers on this subject. Dr. Brack clears up some of the *whys and wherefores* that were so puzzling to the experimenters who, years ago, worked out the results that are accepted without question or thought by the majority of the operators of to-day, and to solve which required practical study of the finer chemical properties of gold, its affinities, etc. Among the substances fatal to the welding property of gold Dr. Black mentions chlorine gas, ammonia, and especially the gaseous compounds of sulphur and of phosphorous which can not be removed by annealing, the welding property being presumably lost. Ammonia affords perfect protection against impurities of the atmosphere and ammoniated foil will be found very convenient for general use; it works perfectly as non-cohesive foil, while its welding property is very perfect and uniform when annealed. Dr. Black finds that the best results in annealing are attained by placing the foil on a mica slab supported over a bunsen gas or alcohol lamp flame, heating the mica nearly to redness, or even to a full cherry red if the gold for any cause does not work satisfactorily. The usual method of annealing piece by piece in foil plyers is open to various objections; phosphorus from the match used in lighting the lamp may cause the ruin of a fine filling; or too much gold is covered by the points of the pliers, which not being heated causes flaking or pitting; or the annealing is done too quickly and the gold not heated through; or on the other hand it reaches the melting point which is even more fatal. A full cherry red gained slowly will give the best results.

Dr. Barrett's method of annealing gold is materially different from that recommended by Dr. Black. He says a gentle heat causes the expansion of metals, a driving asunder of their constituent crystals which are easily forced into a permanent cohesion with each other through an interlacing and interlocking of their granules by means of a sudden impact, as from the blow of a hammer. A very high temperature involves the production of other conditions which are inimical to the desired end. If layers of foil in close proximity are heated to a cherry red, handling with pliers causes a premature cohesion, forming an intractable mass. If layers of thin foil are passed through a flame the corners will curl and melt forming minute lumps fatal to thorough condensation. The best results are obtained only when all the foil is subjected to the same degree of heat. To secure this Dr. Barrett has a batch of foil beaten out by the manufacturer specially for him, and never less than one-quarter pound. If this proves perfectly satisfactory the rest of the batch is secured at once. This is left in the original packages in a dry place secure from atmospheric changes. His apparatus for annealing consists of a tray of the finest thin sheet steel with symmetrical indentations for holding the pellets so that they may not come in contact with each other. Beneath the tray is a small bunsen burner to which it is attached, so arranged as to give an average uniform temperature of about 700° F. (gold at a cherry red being about 2,000°). The annealing tray and burner are permanently attached to the axis of the operating table by a revolving gas-bearing arm, thus bringing the annealing apparatus quite near the patient when in use. The base of the filling and the larger part of the body being made of unannealed foil, the gas is lighted and the gold slowly heated, the transition being gradually made from soft to annealed foil.

### NON-COHESIVE GOLD.

Dr. I. B. Davenport, in the *Cosmos*, gives an illustrated description of his method of using cylinders, or rather half-cylinders and "loops." Williams' cylinders, "style B," are cut in two longitudinally, or cut open with a sharp lancet from circumference to centre, forming a loop permitting every layer of gold to be opened out and flattened against the cavity wall. By this method so favorable to thorough lateral condensation no interstices are left, each layer shingling over free edges of the previous one securing perfect approximation to the wall. Dr. D. thinks that fillings in the grinding surfaces should be made entirely of non-cohesive foil as being more in harmony with the functional requirements of the teeth; they do not produce the uncomfortable sensation of hardness and rigidity so commonly experienced with cohesive work and still more marked with amalgam; they lack the stony hardness and gritty brittleness of the cements and do not produce the dragging leaden sensation of tin or the leathery feel of gutta-percha.

Dr. D. suggests the possibility that capilarity may have something to do with success or failure. In the above method the capillary creeping of fluids between layers of gold arranged concentrically would tend to continually approximate the gold to the walls of the cavity, the moisture in any possible space between the filling and the tooth being thereby diminished the pressure of the inner layers of gold forcing the outer layer against the cavity wall. This may perhaps explain the rapid decay under a leaky cohesive gold filling, capillarity around a stopping of a rigid unchangeable outline acting to the detriment of the tooth.

In the discussion of this paper (which was read before the New York Odontological Society) Dr. C. F. Ives said that he had seen Dr. Davenport insert fillings after this method and was particularly impressed with the ease and rapidity with which they were inserted and their solidity when completed. He had made the most searching tests for imperfections and was dumfounded by the solidity of the structure.

Dr. Brockway said that it struck him as an admirable and philosophic method of manipulating non-cohesive gold; the only wonder being that no one had worked it out before.

Dr. Lord said that fifteen years ago he had learned from Dr.

Dwinelle that "an absolute point" is the point to use, and that he had made better gold fillings from learning that. Dr. Dwinelle brings the instrument down to an absolute point on its four sides at a certain angle and then turns it up at another angle giving it the form of a truncated cone, with which gold can be carried anywhere with perfect security and certainty.

### GOLD INLAYS.

Dr. A. Robinson, in the *Ohio Journal* gives his method of filling large cavities with gold with ease to both patient and operator. The cavity being prepared with walls as nearly perpendicular as may be and without either retaining points or under cuts, a platinum matrix is burnished in very perfectly, great care being taken to have the edges especially well marked. In the laboratory with blow pipe and soldering-block gold is flowed into this matrix until full. If building out is required the matrix is invested in plaster or marble dust and the filling ground on the lathe to the desired size and contour.

Retention is secured by means of a deep groove, giving the filling the appearance of a button stud. The cavity being thoroughly dried is filled with cement and the filling sent home with the mallet.

### CRYSTALLOID GOLD.

Dr. J. G. Reid (*Review*) claims for crystalloid gold, advantages not found in any other form of gold especially in its capability of spreading laterally, rendering it the most suitable for frail cavity walls. When properly annealed it becomes extra-cohesive, it packs very rapidly and evenly giving a surface free from pits or undulations. It should be cut in strips or blocks to correspond with the size of the cavity and introduced with a plugger as large at the point as can be conveniently used in the cavity and not too finely serrated. The best results are obtained by using the gold just as it comes from the manufacturer.

Dr. W. H. Steele (*Items of Interest*) uses William's crystalloid gold or Sibley's felt gold, making the cavity as near a dovetail shape as possible with the bottom as level as it can be made. He has abandoned retaining points owing to the danger of drilling too near the pulp, and also because in forcing gold into the retaining points with the mallet it acts as a wedge causing the tooth to check and crack, ultimately causing the failure of the filling. He finishes with semi-cohesive foil with the mallet and fine points.

Dr. S. G. Perry, in his paper on the Treatment of Proximate Surfaces (*International*, Feb. and Mch.) says of Watt's crystal gold—it stays where it is fixed and is so soft and adjustable and works so beautifully, is used with bell-shaped pluggers, that I often waste time and gold in order to get the fine results that can be had in no other way. It will not, however, give good results unless it is worked with very great care; every piece must be fixed and condensed before another piece is added. When used with a matrix but little finishing will be required. A thin, flat, highly polished burnisher rubbed on a moist piece of soap and carried with a firm hand along the edge of the filling will leave a margin that will make one bless the day when this beautiful form of gold was produced.

### SEPARATIONS.

Dr. G. S. Dean, in the *Cosmos*, discusses the question of separations *versus* contour, taking as a text the paper of Dr. Davenport on The Dental Arches and the discussion of the same in the New York Odontological Society the more recent paper of Dr. J. Morgan Howe on Interdental Spaces, and a certain typical or "extreme" case wherein all the indications are for separation, *viz*:—(1) a systemic predisposition to dental caries due to an uncorrectable fault of the saliva dependent probably upon an error in the generative system; (2) mature age; (3) perfect articulation; (4) jaw of ample size; (5) healthy gingival tissue; (6) absence of the third molars; (7) contact between the teeth with which those to be separated articulate; (8) actual caries on the proximal faces of the teeth to be separated; also, (a) an unfavorable general condition of the patient demanding brief and painless operations; (b) frail tooth structure; (c) limit of dollars.

Of the last three conditions Dr. Dean says—(a) even when local conditions seem to call for restoration of contour separations are best where the general health is unfavorable or when the patient is timid and it is desirable to reduce the operation to the least possible dimensions; less service to the denture being the greater service to the patient. (b) Extensive restoration would be at the risk of devitalization, the choice lying between that and simple conservative filling. (c) Contour operations are relatively expensive, and financial as well as physical conditions must be considered.

Admitting that until "function" has indurated the gingivæ—until the mesial gingival margins have been brought to the level of the buccal and palatine margins as regards sensitiveness, pressure of food between the teeth will be unpleasant and that for effectiveness in cutting and grinding the spaceless denture is superior, he nevertheless claims that separation is a lesser evil than that which it aims to prevent; that as nature has *not* furnished civilized man with either teeth or dental arches "so formed as to be the best" for preventing caries, but *has* conferred on civilized man a special predisponent to caries in the shape of an inefficient and ropy saliva, therefore, they (the separationists) make two much-needed improvements of the poor results of "nature's wisdom;" they close the crown fissure in all cases, and they separate the teeth in those cases which present indications for separation.

### BORDERS

Under this title Dr. L. E. Custer (*Archives*) discusses the conditions and influences which cause recurrence of decay. The line of the edge of the filling next the tooth structure is the critical point and cleanliness the most important factor.

As decay begins where food is held and allowed to decompose, absolute cleanliness must preclude decay. To secure this the edge of the filling should at no place run through those points most susceptible to caries, or in pits and fissures or that point on a proximal surface where the adjoining tooth holds food in contact with it. In molars and bicuspids the border should reach high enough up on the cusps to secure an easily cleansed surface. The border of a filling is the weakest part of a filled tooth and should be dressed down until an even and smooth surface with the enamel is reached. These precautions will insure against recurrence of decay as far as external influences under ordinary circumstances are concerned.

### TIN AND GOLD.

The *Cosmos* publishes a translation of portions of a lecture by Dr. W. D. Miller, of Berlin, on the combination of tin and gold as a filling material, the use of which, Dr. Miller says, lessens the work to both patient and operator, also increasing the permanency of the filling in a way not possible to reach with any other material. This combination was adopted twenty-five years ago by the late Dr. Abbot, of Berlin, father-in-law of Dr. Miller, which may have influenced his earlier use of this material; his continued adherence to it, however, is based on observation and personal experience.

In its preparation Dr. Miller lays a sheet of Abbey's non-cohesive No. 4 gold foil upon a sheet of No. 4 "extra tough" tin foil. The doubled sheets are cut into two or four strips and crumpled into rolls, rolled to the right to a moderately dense rope, then unrolled till it forms a soft crumpled rope. Dr. Miller prefers to have the tin on the outside as being less easily torn; Dr. Jenkins, of Dresden, prefers the gold outside as better illuminating the cavity and retaining better color in the finished filling.

Its desirable qualities are (1) density, becoming in the course of a few weeks as the result of chemico-physical influence so hard that it will resist mastication for years without abrasion, remaining after this change has taken place permanently unaltered. (2) Adaptability; it is remarkably soft and adheres with great ease to the walls of the tooth and a slight expansion which takes place closes hermetically any possible slight defects of contact with the walls of the cavity. (3) It is a poor *conductor* and can be brought nearer the pulp than gold. It has no bad effects on the general health, on the mucous membrane, or on the tooth pulp, and does not discolor dentine. It is not injured by moisture; fillings may be made completely under saliva provided that the cavity has been saturated with an antiseptic. When it is desirable to leave softened dentine over the pulp the first pellets may be saturated with carbolic acid, thus sterilizing the cavity; the dentine becomes denser under such fillings while the hardening of the filling material is accelerated by the moisture. Witzel immerses the first pellets in a phenol-zinc-chloride solution, a more rapid hardening being the result. The mass becomes stone-hard and in cutting is scarcely to be distinguished from amalgam. With very young children, free the cavity from particles of food, remove the external layer of carious dentine, saturate the cavity with carbolic acid, insert the tin-gold and condense; the whole operation in central cavities may be completed in from one to two minutes and will last for years in good condition.

### COPPER AMALGAM.

Dr. St. George Elliott, in a paper read before the Odontological Society of Great Britain, and republished or quoted from, or commented upon in most of the American journals, argues the policy of the generally accepted opinion that copper amalgam does not shrink, and gives the tabulated results of a series of experiments showing comparative shrinkage, daily fluctuation, and breaking strain on edge strength. According to these tests much better results are obtained from silver alloys. This, of course, does not affect its admitted antiseptic value in teeth of poor structure.

Dr. Garrett Newkirk points out in the *Review* some of the difficulties encountered in the manipulation of copper amalgam. He considers the small glass mortars absolutely worthless. The mortar should be of wedge-wood and at least two and one-half inches wide within. Both mortar and pestle should be warmed before using; ingorance or neglect of this one point being the source of much of the unsatisfactory working of copper amalgam. For the same reason it should not be laid on a cold slab, a small porcelain jar lid in which a piece of mica is laid forms a convenient receptacle which can be readily warmed. If too dry do not add more mercury but make it plastic by friction and warmth. If the filling is properly shaped in finishing, so as not to require grinding, the color is much improved by repeated pressings of tin foil on the surface.

Dr. William H. Rollins in a communication to the *Cosmos*, gives the results of his experience in the use of copper amalgam which differ widely from the results generally attributed to this material. He finds that in mouths where decay is not rapid it preserves the teeth about as well as the average gold filling the teeth not staining unless pulpless, in which case discoloration is simply a question of time. When the saliva is abnormal and teeth decay rapidly the amalgam does not turn black, as a rule, but the fillings lose rapidly in weight, to the extent of five per cent. in a year, the surfaces showing a fine crystalline appearance like a fresh fracture of high grade steel. He advises caution in the use of copper amalgam in such cases till it can be proved that the continual introduction of so much copper and mercury into the system is harmless. He also finds and is prepared to prove that copper amalgam changes its shape and therefore does not make a tight filling.

Dr. S. E. Custer, in the *Ohio Journal*, points out the growing necessity for a filling material that shall act as a preservative in teeth of poor structure in these days of increasing degeneracy, and thinks he finds it in copper amalgam. The sulphide of copper which is formed on exposed unamalgamated particles of copper forms the sulphate by oxidation, this layer acting both mechanically and therapeutically—the former by filling any possible gaps with a most compatible layer, and the latter as an antiseptic, preventing fermentation and the growth and action of leptothrix, or bacterum lactis. This position he supports by quotations from Drs. Black, St. George Elliott, and others. The color only prevents a more extended use of copper amalgam. Dr. Custer says that this has also edge strength and slow or quick setting are all within control by the manner of manipulation. The setting time is in inverse ratio to the amount of heat applied. All grades between the two extremes being attainable at will. Too much heat is the cause of the objectionable blackening, mercury being vaporized in such quantities that free surfaces of copper are left which do not again become amalgamated and which forms the black salts.

If the amalgam is triturated in a weak solution of mercuric nitrate (one to five of water) a solution of bicarbonate of soda being added to neutralize the acid, the mass readily becomes plastic and the resultant filling whiter, though, perhaps at the expense of the saving power of the amalgam through the prevention of the formation of the copper salts whose virtues we desire while we object to the color they give.

In the New York Odontological Society, reported in *Cosmos*, Dr. S. E. Davenport described a case in which the six year molars of a lad eight years old were badly decayed, the tooth structure having the feel of chalk or hard cheese. Removing only that which was exceedingly soft, leaving much that was discolored and apparently breaking down; the teeth were filled with copper amalgam. Six months later an almost miraculous change had taken place. The margins were almost perfect and the tooth substance quite hard with the ringing sound of at least a fair quality of tooth structure. After four years the teeth are still being preserved by these fillings. Dr. Davenport does not find that copper amalgam discolors dentine much more than other amalgams and he does not hesitate to use it where the necessity for its saving qualities is apparent.

Dr. Southwick has used copper amalgam for the last year with great satisfaction.

Dr. S. S. Straw has almost entirely discarded all other amalgams in favor of copper.

The Committee on Dental Practice (see N. Y. Trans.) recommends packing amalgam in small masses with the rotary motion, and when sufficient amalgam is inserted taking small pieces of gold foil No. 1, and after annealing burnish them lightly over exposed portion of the filling; the gold will be entirely taken up by the excess of mercury and disanpear leaving perfect edges and finish, the color of bright steel.

Dr. R. B. Adair (*Archives*) gives some practical hints about amalgam. The simple test for shrinkage is a properly prepared filling in one end of a glass tube, the balance of the tube filled with an analine solution; if none goes down between the filling and the glass wall there is no shrinkage.

An amalgam that does not darken within one hundred hours in a solution of sulphate of potash (60 grains to the ounce) will retain a good color in the mouth. Dr. Adair says amalgam is established on a firm basis beyond the reach of any society or organization ; it has come to stay.

"A," in the *Southern Dental Journal*, January, advocates earnestly the more frequent use of amalgam. He says very truly "a tooth well preserved with amalgam is worth as much to its possessor as if filled with more costly material." That this may be the case the cavity must be as thoroughly prepared, the material inserted with as much care, and the finish be as fine as if gold were used. In the preparation of amalgam for use the mercury must be so thoroughly incorporated that the mass may have a velvet-like feel. After adding a few drops of sulphuric acid it should be well washed with water and alcohol and squeezed in twisted chamois skin until no visible globules of mercury pass through; any excess of mercury in the filling may be removed by the application of pads of tin foil. The surface should be shaped with points of soft wood carrying pulverized pumice. The preservation of the tooth, not the material used, is the thing appreciated by thinking patients.

In the *Archives* of February Dr. L. C. Ingersoll emphasizes the importance of thoroughly washing amalgam in order to remove the oxides, a dark colored powder which gives the mass a dirty grayish look and not combining with the other ingredients, dissolves from the surface of the filling and renders it porous.

In the *Dental Record* (London) Dr. Thos. Fletcher criticizes Dr. Ingersoll's statements rather severely. He says that Dr. I. calmly ignores any tests and makes statements as to what is washed away from an amalgam without the slightest basis for his statements as he evidently has made no analysis. Dr. Fletcher says that what can be washed away from an amalgam depends largely on the state of division of the metal and the chemicals used to produce the discoloration which is to be washed away.

Dr. B. R. Stevens washes the contents of a fresh package of amalgam three or four times in alcohol, or until the latter is not discolored. After drying on a clean piece of paper it is kept in open-mouthed vials which Dr. S. thinks causes it to set more evenly and slowly, new amalgam setting too quickly and working "harsh." He burnishes the amalgam.

